# Molecular mechanisms of sex bias differences in COVID-19 mortality

**DOI:** 10.1186/s13054-020-03118-8

**Published:** 2020-07-09

**Authors:** Yuchong Li, Mirjana Jerkic, Arthur S. Slutsky, Haibo Zhang

**Affiliations:** 1 The State Key Laboratory of Respiratory Disease, Guangzhou Institute of Respiratory Disease, The First Affiliated Hospital of Guangzhou Medical University, Guangzhou, Guangdong China; 2 The Keenan Research Centre for Biomedical Science, St. Michael’s Hospital, Toronto, Ontario Canada; 3 Interdepartmental Division of Critical Care Medicine, University of Toronto, Toronto, Ontario Canada; 4 Department of Anesthesia, University of Toronto, Toronto, Ontario Canada; 5 Department of Physiology, University of Toronto, Toronto, Ontario Canada

**Keywords:** SARS-CoV-2, Hormone, Chromosome, Estrogen, Androgen, Immune response

## Abstract

More men than women have died from COVID-19. Genes encoded on X chromosomes, and sex hormones may explain the decreased fatality of COVID-19 in women. The angiotensin-converting enzyme 2 gene is located on X chromosomes. Men, with a single X chromosome, may lack the alternative mechanism for cellular protection after exposure to SARS-CoV-2. Some Toll-like receptors encoded on the X chromosomes can sense SARS-CoV-2 nucleic acids, leading to a stronger innate immunity response in women. Both estrogen and estrogen receptor-α contribute to T cell activation. Interventional approaches including estrogen-related compounds and androgen receptor antagonists may be considered in patients with COVID-19.

## Background

The COVID-19 pandemic has led to hundreds of thousands of deaths worldwide. In South Korea, men were far less likely (~ 38%) to be diagnosed with COVID-19, but their mortality rate was about twice that of women (1.2% for men versus 0.5% for women) [1]. In a European study, males had a slightly higher incidence of COVID-19 with no respiratory failure than females (57.7% versus 42.3%), but many more males with COVID-19 developed severe respiratory failure compared to females (89.3% versus 10.7%) [2]. Most studies have demonstrated that males are more likely than females to die from the SARS-CoV-2 infection across all age groups under 90 years [1, 3, 4]. This same pattern was apparent during the SARS outbreak in 2003: males had a significantly higher case fatality rate than females (21.9% versus 13.2%); the relative risk was 1.66 and was 1.62 after adjustment for age [5].

Is this phenomenon biological, some quirk of cells and hormones? Is it the result of gendered behaviors? There are a number of hypotheses to explain different responses to SARS-CoV-2, including a number of social and cultural differences between males and females, such as smoking history, differences in underlying diseases [6], or biological differences due to sex-specific immune defense factors [7]. For example, male mice are more vulnerable to SARS-CoV infection compared with age-matched females [8], and this relationship increases with increasing age. Middle-aged mice had higher viral titers, elevated vascular leakage, and alveolar edema compared with young mice, but blocking estrogen in female mice was associated with greater lung injury and a higher mortality rate [8].

In this viewpoint, we discuss the impact of sex on the immune response focusing on the X chromosome and female sex hormones, which may provide clues for potential mechanisms that may lead to new and effective treatments for COVID-19.

### Are there chromosome-bias differences in angiotensin-converting enzyme 2 (ACE2) activity?

ACE2 is a major link in the pathogenesis of COVID-19 and has two major biological functions in this context: (1) it catalyzes the conversion of Ang I and Ang II to Ang 1–9 and Ang 1–7, respectively, which leads to organ protection, and (2) serves as the receptor for the entry of SARS-CoV-2 into cells [9].

A total of 16 residues of the SARS-CoV-2 receptor binding domain contact 20 residues of ACE2, and there are some different ACE2 interactions both in and outside the SARS-CoV-2 receptor binding motif (RBM) [10].

The ACE2 gene is located on X chromosomes. Generally, one female X chromosome is randomly inactivated leading to equal gene and protein expression between sexes, a process called X-inactivation [11]. However, the ACE2 gene location on Xp22.2 is an area where genes avoid this X-inactivation [12], contributing to phenotypic differences between sexes [13]. The location of the ACE2 gene on the X chromosome is important for its ability to recognize the SARS-CoV-2. Since SARS-CoV-2 carries 16 residues of RBM, they bind to 16 out of 20 residues on ACE2 in males [10]. The same SARS-CoV-2 RBM can be recognized by ACE2 on either of the two X chromosomes in females. The chance that the same residue sequences of ACE2 on the second chromosome also perfectly binds to the SARS-CoV-2 RBM is low, allowing unbound ACE2 to catalytically cleave Ang II to form Ang 1–7, and thus could decrease the likelihood of pulmonary edema during COVID-19 [14]. This mechanism is specific to females because the two X chromosomes function in a coordinated fashion. Men, with a single X chromosome, lack the alternative mechanisms that could lead to cellular protection during COVID-19. However, there are no data so far reporting the expression levels of ACE2 and Ang II in patients with COVID-19.

The location of the ACE2 gene on the X chromosomes may also play a role in the different prevalence of cardiovascular disease in COVID-19 between males and females [15]. A murine model demonstrated that intranasal inoculation with SARS-CoV led to marked myocardial infection and damage and decreased myocardial ACE2 expression [16]. In humans, the SARS-CoV viral RNA was detected in 35% of autopsied heart samples during the SARS outbreak in Toronto [17]. The downregulation of X-linked ACE2 in cardiovascular tissues after binding to SARS-CoV-2 [16] could be more harmful in males than females alongside ACE2 gene mapping on X chromosomes.

### Are there chromosome bias differences in pattern recognition receptors (PRRs)?

Toll-like receptors (TLRs) are PRRs that recognize pathogen-associated molecular patterns (PAMPs) from viruses to initiate innate immune responses. Several TLR signaling genes including TLR3, TLR4, and TLR7 are encoded on the X chromosomes [18, 19]. TLRs sense double-stranded RNAs, a transitional nucleic acid species active during SARS-CoV infections [19]. TLR3 and TLR4 individually mediate a portion of the Toll-interleukin-1 (IL-1) receptor domain-containing adaptor inducing interferon-β (IFN-β) [TRIF]-dependent signaling, contributing to protection from SARS-CoV [19]. Another common TLR4 pathway involves signaling via IL-1 receptor-associated kinases (IRAKs) and inhibitors of nuclear factor kappa-light-chain-enhancer of activated B cells (NFκB) kinases (IKKs). IRAK1 and IKKγ are encoded on the X chromosome at Xq28 [18] may confer an advantage in responding to and resolving SARS-CoV-2 infections in females [20].

Dendritic cells (DCs) utilize TLR7, encoded by a gene on the X chromosomes, which can sense SARS-CoV-specific GU-rich single-stranded RNA [21]. High copy numbers of TLR7 and elevated interferon regulatory factor-7 (IRF-7) expression induce robust interferon-γ (IFN-γ) expression, leading to a greater protection against human immunodeficiency virus infection in women [22]. Excellent review articles elsewhere have discussed emerging studies exploring the influence of TLR7 on sex-specific immune responses [23]. The finding that the X chromosome contains 112 micro-RNAs (miRNAs), compared with only 2 miRNAs on the Y chromosome [24], is very interesting and may suggest that these PPR genes contribute to sex differences in COVID-19, including after menopause.

Recent studies have demonstrated that the X-chromosomes together with sex hormones act in concert to increase the production of IFNs by dendritic cells contributing to the enhanced TLR7-mediated response in females [23]. This finding may have implications for our understanding of the pathogenesis of sex differences in COVID-19 [1, 3, 4].

A cytokine storm has been reported in patients with severe COVID-19 [25]. This inflammatory response is often considered to be a double-edged sword because it is required for the control and elimination of SARS-CoV-2. However, an overwhelming cytokine storm may lead to organ failure and death via cross-talk between PRRs and PAMPs. In males, the cells occupy a PRR gene’s locus that may be adequate, hypo- or hyperresponsive to SARS-CoV-2 infection. In females, the two sets of cells varying in X-linked polymorphic immune-competent genes with different X chromosome regulatory and activation capacity are unique [26], which may suggest a more balanced and more adaptive system during the cytokine storm in COVID-19.

### Are there sex hormone bias differences in organ protection?

Estrogen is a powerful steroid involved in numerous biological processes in a variety of tissues. There are three different types of estrogen: (1) Estrone (E1) a major hormone found in high quantities in postmenopausal women and produced primarily by the ovaries. Estrone is the least powerful of the three types of estrogen [27]. (2) 17β-estradiol (E2) produced mainly in the ovaries and also synthesized in the brain and adrenal glands, and the testes in men [28]. Females have much higher levels of this hormone than males, and it is the most common type of estrogen in female reproductive years. Estradiol is the most potent of the three estrogens. (3) Estriol (E3) produced mainly by the placenta. Levels of estriol in the body are very low but higher during pregnancy.

There is a growing interest in the regulation of the tissue renin-angiotensin system (RAS) by sex hormones. 17β-estradiol regulates the expression of ACE2 in the heart, kidney, and uterus [29, 30]. For example, 17β-estradiol increases local ACE2 activity in the heart and attenuated the RAS system by cleavage of a single residue from Ang II to enhance the production of cardioprotective Ang 1–7 and promote anti-inflammatory and anti-oxidative effects [31–33].

Estrogen levels inversely correlate with the expression of cardiac troponin (cTn), released from cardiomyocytes exposed to ischemia or hypoxia causing irreversible cardiac damage [34]. In COVID-19 patients, studies have found that 51% of patients with cardiac injury died versus 4.5% who did not have it [35]. The mortality rate was 7.6% in COVID-19 patients without underlying cardiovascular disease (CVD) and normal cardiac troponin (cTn) levels, 13.3% in patients with underlying CVD and normal cTn levels, 37.5% in patients without underlying CVD but elevated cTn levels, and 69.4% in patients with underlying CVD and elevated cTn levels. A higher proportion of men (65.4%) had elevated cTn compared to women (42.2%) with COVID-19 [35]. The action of estrogen may explain these findings, since estrogen has been shown to decrease low-density lipoprotein (LDL) cholesterol concentrations, increase high-density lipoprotein (HDL) [36]. The 17β-estradiol in particular mediates early and late endothelial nitric oxide synthase (eNOS) activation through interaction with estrogen receptors. Functional estrogen receptors are also present in cardiomyocytes, which regulate the expression of NOS to protect against cardiovascular injury by inhibition of platelet activation, thrombus formation, and leukocyte-endothelial cell adhesion [37].

Emerging evidence shows that patients with severe COVID-19 can have a coagulopathy characterized by hypercoagulability with fibrin formation and polymerization leading to thromboembolism and worse outcomes, especially in men compared to women [38]. This might be partially explained by the fact that estrogen can reduce plasma levels of fibrinogen and increase antithrombin III [39] and decrease plasminogen-activator inhibitor type 1 (PAI-1) [40].

A reasonable question is that if the mechanism of organ protection is female sex hormones, why is there still a difference in COVID-19 mortality in postmenopausal women? In premenopausal women, the ovaries are the principal source of 17β-estradiol. In postmenopausal women, estrone, the least powerful form of estrogen, is produced in the ovaries in high quantities. Moreover, in postmenopausal women, and in men, estrogen is no longer solely an endocrine factor. Instead, it is produced in a number of extragonadal tissues and acts locally at these sites as a paracrine or even intracrine factor. These tissues include the mesenchymal stromal cells of the adipose tissue and subsequent differentiated bone chondrocytes and osteoblasts, aortic and endothelium, vascular smooth muscle cells, skin, skeletal muscle, and numerous brain regions [27]. Thus, circulating levels of estrogen reflect estrogen action in postmenopausal women and in men as a result of the estrogen that escapes from local metabolism and enters the circulation after production by extragonadal tissues [27]. Despite low circulating levels, high local concentrations indicate the potential of paracrine, autocrine, and intracrine actions of estrogens in tissues [27]. However, it is unknown whether the distribution and expression of estrogen in the local tissue contribute to the lower COVID-19 mortality in postmenopausal females compared to males.

### Are there sex hormone bias differences in immune response?

The observation of the higher lethality of SARS-CoV-2 in males may be partially related to sex-specific immune responses. In general, the number, development status, and function of immune cells differ markedly between the sexes in humans; with immune cells obtained from females generally exhibiting a stronger response than cells isolated from males. For example, a greater number of residual macrophages are observed in the alveolar space and peritoneal cavities of female mice compared to male mice; and female macrophages undergo phagocytosis more efficiently and express higher levels of TLR4 compared to male resident macrophages [41]. Females have higher basal immunoglobulin levels, larger numbers of circulating and resident CD4/CD8 T-lymphocyte ratio than males [41, 42].

Sex hormones influence the repertoire of the immune responses differently in men and women. Both estrogen and estrogen receptor-α contribute to T-lymphocyte activation and proliferation and induce high IFN-γ in T-lymphocytes. A delayed IFN-α response was accompanied by vigorous SARS-CoV replication in male mice [43]. Researchers have demonstrated that IFN-β and IFN-γ can potently inhibit SARS-CoV replication, and a synergistical anti-SARS-CoV effect was achieved with the combination of IFN-β and IFN-γ [44]. In animal experiments, estrogen treatment silences the inflammatory reactions and decreases SARS-CoV titers leading to improved survival rate [8].

Unlike estrogen, however, testosterone has been shown to have an overall suppressive effect on the immune system, in particular to viral and host antigens [45]. For example, the treatment of mouse macrophages with testosterone inhibited NOS [46]. Androgens may repress the proliferation of thymocytes by inducing thymic apoptosis [47]. Studies have demonstrated a strong immune-suppressive role of testosterone on conventional dendritic cell (cDC) activation, presentation of antigen to T-lymphocytes and induction of protective immune response against pathogens [48]. T-helper cells such as Th1 cells play a critical role in protecting patients from viral infections by producing IFN-γ, promoting innate and adaptive immune responses for the clearance of pathogens [49]. Androgens can alter T-lymphocyte immunity by inhibiting Th1 differentiation and decreasing IFN-γ production [50]. Overall, androgens can affect T-lymphocytes both directly and indirectly by modulation of Th1 cell differentiation and inhibition of the necessary Th2 response required to clear the infection, thereby suppressing general immune functions, which may explain the high fatality of SARS-CoV-2 infections in men [1–4].

## Conclusions

**Fig. 1:**
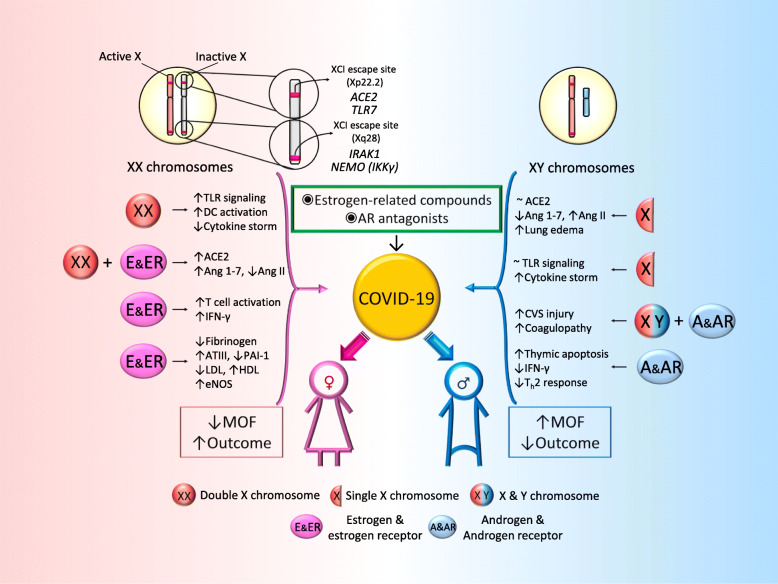
Main mechanisms responsible for increased immune activity in females, providing a survival advantage in COVID-19. In females, the second X chromosome is randomly silenced during X chromosome inactivation (XCI) in order to minimize duplication of proteins encoded by X-linked genes. However, several genes bypass this inactivation. The ACE2 gene location on Xp22.2 is known to avoid X-inactivation, contributing to phenotypic differences between sexes. IRAK1 and IKKγ are also encoded on the XCI escaping sites, which may confer an advantage in responding to and resolving SARS-CoV-2 infections in females. The X chromosome has numerous genes involved in immunity. Naturally occurring variations in one gene copy might result in two distinct alleles with different regulatory and response capacities, suggesting that females, but nor males, may not only avoid the effects of deleterious gene mutations, but also benefit from added physiological diversity when facing new immune challenges, such as SARS-COV-2 infections. Estrogen and estrogen receptor signaling play a crucial role in both innate and adaptive immune responses as well as in tissue repairing processes during respiratory virus infection. Sex-based targeted therapeutic and interventional approaches such as estrogen-related compounds and androgen receptor antagonists may be considered in patients with COVID-19. ACE2: angiotensin-converting enzyme 2; TLR7: Toll-like receptor 7; IRAK1: Interleukin-1 receptor-associated kinase 1; NEMO (IKKγ): NF-kappa-B essential modulator (inhibitor of nuclear factor kappa-B kinase subunit gamma); DC: dendritic cell; TLR: Toll-like receptor; Ang 1–7: angiotensin 1–7; Ang II: angiotensin II; IFN-γ: interferon gamma; ATIII: antithrombin III; PAI-1: plasminogen activator inhibitor-1; LDL: low-density lipoprotein; HDL: low-density lipoprotein; eNOS: endothelial nitric oxide synthase; CVS: cardiovascular system; T_h_2: Type 2 helper T cells; MOF: multiorgan failure

The sex bias in COVID-19 deaths may provide clues to mechanisms of injury. These findings may be due to known sex differences in genes, chromosomes, and hormones that lead to very different responses to many diseases, including COVID-19 (Fig. 1). We suggest that sex should be taken into account when designing and analyzing clinical trials in both animal and human studies in SARS-CoV-2 infection and that sex differences may reveal novel therapeutic and interventional approaches such as estrogen-related compounds and androgen receptor antagonists in the treatment of patients with COVID-19.

## Data Availability

Not applicable
